# SARS-CoV-2 Psychiatric Sequelae: An Urgent Need of Prevention

**DOI:** 10.3389/fpsyt.2021.738696

**Published:** 2021-09-09

**Authors:** Hugo Bottemanne, Fanny Delaigue, Cédric Lemogne

**Affiliations:** ^1^Paris Brain Institute—Institut du Cerveau (ICM), UMR 7225/UMR_S 1127, Sorbonne University/CNRS/INSERM, Paris, France; ^2^Sorbonne University, Department of Philosophy, SND Research Unit, UMR 8011, Paris, France; ^3^Sorbonne University, Department of Psychiatry, Pitié-Salpêtrière Hospital, Assistance Publique-Hôpitaux de Paris (AP-HP), Paris, France; ^4^Université de Paris, AP-HP, Hôpital Hôtel-Dieu, DMU Psychiatrie et Addictologie, Service de Psychiatrie de l'adulte, INSERM, Institut de Psychiatrie et Neurosciences de Paris (IPNP), UMR_S1266, Paris, France

**Keywords:** COVID-19, COVID-19 survivors, psychiatric sequelae, coronavirus, mental health, public health, depression, anxiety

## Abstract

Severe Acute Respiratory Syndrome Coronavirus 2 (SARS-CoV-2), responsible for COVID-19 pandemic, caused catastrophic health and social effects, but little is known about its consequences on mental health. Other viral infections have been associated with psychiatric sequelae: infection-triggered disturbing of the immune system and the stressful intensive unit care can cause psychological and psychiatric complications. Moreover, SARS-CoV-2 can potentially induce neuronal injuries, leading to neurocognitive disabilities. Previous studies during the COVID-19 pandemic reported a high occurrence rate of psychopathological and neurocognitive conditions among COVID-19 survivors, highlighting the need for screening for these impairments in order to implement early interventions and secondary prevention. However, many psychiatric disorders can take several years to develop, and it is still difficult to differentiate between factors linked to the infection itself or to the global context of the pandemic. In this review, we describe the effects of SARS-CoV-2 infection on mental health, the mechanisms involved in psychiatric and neurocognitive sequelae, and the strategies of prevention and management. More studies are needed to investigate the effects of a range of factors including clinical, sociodemographic, and inflammatory predictors. These efforts could be useful to identify high-risk individuals and inform targeted preventive actions.

## Introduction

On January 2021, the outbreak of Severe Acute Respiratory Syndrome Coronavirus 2 (SARS-CoV-2, responsible for COVID-19 disease) has affected more than 77 million of people around the world according to the World Health Organization (WHO). Although most individuals infected by SARS-CoV-2 seem to be asymptomatic or experience mild symptoms, infection may cause severe acute respiratory distress, and an excessive host immune response leading to an inflammatory and procoagulant state responsible of multiple thrombosis and premature death (1). Approximately 20% of infected individuals develop severe illness that requires hospitalization, and 25% of hospitalized patients require a stay on an intensive care unit (ICU) (2). Most of researches have studied mental health among health care providers involved during COVID-19 crisis (3–6), in general population (7–11), or for patients with pre-existing psychiatric disorders (12, 13). Yet, little work has focused on psychiatric and neurocognitive consequences in survivors of SARS-CoV-2 infection.

The association between psychiatric pathologies and RNA viruses, especially coronaviruses, is well-established. Prior studies of patient survivorship after infections by two other coronaviruses, SARS-CoV-1 and MERS-CoV (responsible for Middle East Respiratory Syndrome), as well as an orthomyxovirus, influenza virus H1N1, have found both acute and long-lasting psychopathological consequences (14). In the immediate aftermath of these epidemics, studies have shown that SARS survivors reported high prevalence of depression, anxiety, and post-traumatic stress disorder (PTSD) symptoms (15–17).

In line with these previous data, current reports suggest that SARS-CoV-2 infection seems to be associated with neuropsychiatric consequences (18, 19). These studies are mainly retrospective, and focused on the early effects of the infection, from few weeks to few months. They cannot accurately predict the global severity of the pandemic's effect on mental health, but they can give us some clues about the immediate effects of SARS-CoV-2 infection, and allow us to explore the role of the neuroinflammation in psychiatric disorders. We offer here a review of the effects of SARS-CoV-2 infection on mental health, the mechanisms involved in these effects, and the strategies of prevention and management.

## SARS-CoV-2 Consequences for Mental Health

A first study comparing the mental status of 103 hospitalized patients tested positive with SARS-CoV-2, matched for age, gender, education level, and place of residence—but not for current hospitalization—with 103 healthy control patients recruited from local communities, has shown that COVID-19 patients exhibited higher levels of depression, anxiety, and PTSD symptoms (20). Moreover, another study screening psychological distress with self-rating scale among 126 COVID-19 survivors in early convalescence found a significant proportion of depression (38.1%), PTSD (31.0%), and anxiety (22.2%) symptoms (21). Likewise, a study evaluating psychiatric symptoms in 402 COVID-19 survivors at 1 month follow-up after hospitalization also found a significant proportion of anxiety (42%), insomnia (40%), depression (31%), PTSD (28%), and obsessional-compulsive symptoms (20%) (22).

These three studies found few predictors significantly associated with a higher probability of developing one or more of these psychiatric illnesses, such as having family members infected by SARS-CoV-2 or a post-infection physical discomfort, including gastrointestinal and respiratory symptoms (20–22). Lately, a large-scale retrospective study based on electronic health records showed that incidence of any psychiatric disorder up to 90 days after a COVID-19 diagnosis was about 18%, of which 5.8% were first diagnoses. These first psychiatric cases were more frequent after COVID-19 diagnosis that after several other health events such as respiratory and skin infections, lithiases, or large fractures. Among newly diagnosed psychiatric illnesses up to 90 days after SARS-CoV-2 infection, most prevalent ones were anxiety disorders (4.7%), mood disorders, especially depression (2 and 1.7% respectively), insomnia (1.9%, of which 60% were not associated with anxiety) and dementia (1.6% of patients aged 65 years old and above) (23).

## Biological and Psychological Mechanisms Involved

Should causal relations be involved, mechanisms involved in neuropsychiatric conditions after SARS-CoV-2 infection remain unclear. Here, we suggest that at least four components may explain the link between SARS-CoV-2 infection and poor mental health: somatic symptoms and medical care, immune response, neurocognitive effects, and psychosocial conditions. Preliminary reports suggest that COVID-19 symptoms *per se*, such as dyspnea and anosmia, but also stressful medical care in ICU are major causes of anxiety. Moreover, social isolation and uncertainty of the future may induce or worsen neuropsychiatric conditions following the infection (14, 19, 20, 24). However, biological links between SARS-CoV-2 and mental health must not be ignored: direct neuronal injuries of the central nervous system, immune-inflammatory activation inducing thromboses and vascular damages, and their residue following recovery could have key implications for these psychiatric disorders. Currently, effects of these immune responses and inflammatory sequelae remain unclear.

Survivors of intensive care are known to be at higher risk of developing psychiatric symptoms, especially after acute respiratory distress syndrome (ARDS) (25). Approximately 80% of patients surviving acute respiratory failure after receiving mechanical ventilation develop a post-intensive care syndrome (PICS), defined as a deteriorated health condition including cognitive and psychological impairment (26). A large multicenter study assessing psychiatric disorders in the first year following discharge from an ICU has found a significant proportion of anxiety (46%), depression (40%), and PTSD (22%) (27), and that these symptoms might persist up to 2 years following hospital discharge (28). These illnesses have been associated with comorbid cognitive sequelae such as impairments in attention, memory, and executive function. Likewise, in Wuhan, 40 to 88% of severe COVID-19 patients displayed neurological symptoms, associated with neuroinflammation, demyelination and neurodegeneration such as acute cerebrovascular diseases or conscious disturbance (29). These data related to ICU treatment are difficult to separate from the biological consequences of the infection, in particular the inflammatory and neurotoxic role. Most of patients who end up in ICU have had more severe forms, with a multitude of heterogeneous factors involved in the pathogenesis of psychiatric disorders.

## A Cytokine Storm in the Brain

Emerging evidences suggest that SARS-CoV-2 infection can cause a so-called “cytokine storm”, i.e., local and systemic production of cytokines, chemokines, and other inflammatory mediators (18). Indeed, studies show that SARS-CoV-2 induces high levels of Interleukin (IL)-1β, IL-6, Interferon (IFN)-γ, CXCL10, CCL2, and T-helper-2 cell-secreted cytokines such as IL-4 and IL-10 (30). These highly expressed cytokines may have a range of side effects, such as a disturbing of hypothalamo-pituitary-adrenal and neuroendocrine axes, thus further compromising host's immunocompetence (31). Many studies have pointed out the role of immune system perturbation and neuroinflammation in the rise of psychiatric illnesses, and of high systemic inflammation in mood, anxiety, and psychotic disorders (32–34).

Some previous results suggest that psychological distress is related to inflammation during SARS-CoV-2 infection. For instance, the levels of CR, a peripheral inflammatory indicator, were positively correlated with the levels of depressive symptoms, as assessed with the 9-item Patient Health Questionnaire, in COVID-19 survivors (20). Furthermore, baseline systemic immune-inflammation index, representing the immune response and systemic inflammation, was independently positively associated with scores of depression and anxiety at 1 month follow-up (22). These data are still difficult to analyze, especially since these various studies do not find stable and replicable inflammatory biomarkers that could explain the causal link between inflammation and psychiatric disorders. There appears to be a heterogeneity of inflammatory response, and the numerical value of systemic markers of inflammation may be insufficient to assess their pathogenic role. Likewise, the post-sepsis syndrome, i.e., neurocognitive impairment and deteriorating medical conditions in sepsis survivors, may worsen mental health conditions in these patients in the same way as in the post-intensive care syndrome PICS in which most of patients had sepsis (35).

## A Neurotropic Virus

In addition, pre-clinical studies have demonstrated that coronaviruses are neurotropic, and thus are able to induce neuronal injuries (36). Indeed, neuronal cells expressing ACE2 protein, known to be one of the receptors used by SARS-CoV-2 to infect cells, have been involved in host viral invasion. SARS-CoV-2 can reach the nervous system through several pathways from peripheral nerve fibers of the pulmonary network and the enteric nervous system, as well as using the lymphatics and lymphoid tissues, and also by the olfactory cells of nasal epithelium. Moreover, systemic inflammation and mechanical ventilation in ICU may weaken blood-brain barrier, majoring cytokine-mediated brain damage and the hematogenous dissemination of SARS-CoV-2 in the brain (37).

Neurological damages are caused by this combination of cerebral hypoxia related to viral infection and virus-induced inflammatory responses running such as phagocytosis and cytotoxic mechanisms from microglial cells. More particularly, dysosmia, which is a clinical hallmark of COVID-19 disease in 56% of patients, has been related to viral proliferation in the olfactory bulb (38): progressive inflammation of the olfactory epithelium can lead to alterations of olfactory receptors expression or even to olfactory cells death, and thus to the disrupting of olfactory bulb functions. Knowing the potential role of the olfactory bulb in depressive disorders development (39), potential long-standing effects should be evaluated.

## Psychological and Environmental Stressors

Finally, viral infection produces significant psychological and environmental stressors that may intermingle with psychopathological outcomes, such as uncertainty of future and social isolation experienced by infected patients (40, 41). Indeed, families are prohibited from visiting the hospital during acute infection, and health-care staff reduces their human interactions, especially with infected patients. Isolated ones may experience loneliness, boredom, fear and anxiety concerning their health or socioeconomic conditions (42).

Among these components, several factors such as preexisting psychiatric disorders or physical frailty, as well as social inequalities may worsen recovery from critical illness due to SARS-CoV-2 (43). Studies display that patients with pre-existing psychiatric diagnosis suffered more from psychiatric consequences of SARS-CoV-2, despite a similar baseline of inflammation markers compared to initially healthy patients (22). In another previous study, being female have been associated with PTSD conditions, whereas survivors aged above 60 years old experienced less severe stress response symptoms, emotional symptoms of depression and anxiety than younger survivors (21). These results in older patients may seem paradoxical given the severity of the infection in this population and should be explored by further studies.

## From Risk Factors to Prevention Strategies

These preliminary studies allow us to identify some risk factors that could inform secondary prevention strategies such as targeted screening. In particular, presence of previously diagnosed psychiatric disorder is probably one of the major factors associated with neuropsychiatric features after SARS-CoV-2 infection, and these complications should be systematically sought out in cured patients. Furthermore, the presence and severity of physical sequelae concerning the respiratory or digestive tracts also appears to be one of the main factors of psychiatric sequelae: personalized support by a psychotherapist should be systematically offered to all patients who retain physical sequelae after COVID-19 recovery. Finally, several studies highlight the higher prevalence of psychiatric sequelae in women than in men, despite lower rates of inflammatory markers and less severe clinical forms than them. This sex-dependent association deserves further research to identify underlying mechanisms that could be the targets of preventive actions.

However, there is a current knowledge gap between this preliminary data, and the information needed to develop primary prevention strategies. These studies do not always make it possible to formally identify the factors that are significantly associated with a risk of developing psychiatric disorders after COVID-19, and also show some inconsistencies. For instance, some studies have found that hospital stay length is negatively correlated with the risk of developing a psychiatric disorder (depression, anxiety disorder, and PTSD), while other ones have highlighted the impact of medical treatment on the risk of psychiatric sequelae development. Although plausible, the extent to which age and sex differences may account for these inconsistencies—older age and male sex being both risk factors for severe COVID-19 and protective factors for subsequent anxiety and depression symptoms—remains to be determined. Indeed, despite generally less severe SARS-CoV-2 infections, associated with few physical symptoms and limited inflammatory consequences, young people display a high prevalence of psychiatric sequelae. These paradoxes invite promising avenues of research in order to differentiate the socio-affective and immuno-inflammatory dimensions of risk factors for psychiatric sequelae, not only in retrospective studies like most of studies available for now but also with prospective studies.

Furthermore, multicentric studies with larger cohorts are needed to also better understand to what extent uninfected people with or without known risk factors are concerned by the development, the exacerbation or the upsurge of psychiatric pathologies. Most of previous studies have been carried out in economically-developed countries in which psychiatric disorders are more studied and often early treated. Future studies will have to be led in less developed regions, equally affected by SARS-CoV-2, but with more fragile or less suitable health systems. These needed studies are likely to show us that the currently visible consequences of SARS-CoV-2 pandemic are only the tip of the iceberg when it comes to worldwide mental health.

## Early Strategies are Needed

Despite the lack of established specific risk factors of psychiatric sequelae, these previous results suggest that SARS-CoV-2 infection may have a substantial and detrimental impact on mental health and highlight the urgent need to establish more personalized preventive protocols. Survivors exhibited a high prevalence of neuropsychiatric symptoms, and these symptoms could have a lasting influence on individuals' quality of life, not to mention the burden this would place on psychiatric care. But many psychiatric disorders may also take several years to develop, and early detection could underestimate the overall incidence of these illnesses. Aversive events can contribute to the weakening of individuals' mental health and produce pathogenic effects well after their occurrence, in particular in the field of mood disorders. Indeed, only few studies about COVID-19 consequences have focused on long-term psychiatric sequelae, and unfortunately the hindsight we have about them is only of 1 year: we are likely to underestimate the long-term consequences as some psychiatric illnesses could appear years after infection or last for years.

Early intervention, but also more delayed ones, appears crucial to reduce psychological distress associated with avoidable risk factors such as medical care and psychosocial conditions. A first line of primary prevention should be set up with a systematic screening of pre-existing psychiatric disorders and identified risk factors, to promote early interventions in at-risk infected patients. As we have shown, more research is still needed to better identify individuals risk factors and to guide screening. A psychiatric consultation could be systematically integrated during hospital care for COVID-19, and telephone consultations could be organized for at-home patients. For at-risk individuals, some preliminary studies recently suggested that selective serotonin reuptake inhibitors (SSRI) may have protective properties against severe COVID-19 episodes, while their use could be beneficial for patients with anxiety or depressive risk factors (44, 45). Although further studies are needed to confirm this observation, the adjunction of an SSRI during COVID-19 disease management may not cause harm (45). This first line should be reinforced with secondary prevention by screening cured patients for all prodromal neuropsychiatric sequelae, in particular after severe immuno-inflammatory COVID-19 forms, or after a switching to ICU. A specialized consultation at 2 weeks and 1 month with a psychiatrist should be offered for all patients who have had a moderate to severe form of COVID-19.

## Conclusion

Regarding the biological risks associated with inflammation and neuronal injuries, more studies are needed to assess the pathophysiology of these neuropsychiatric conditions. These monitoring protocols should be offered over the long term, particularly in patients at risk of developing a psychiatric disorder, or for whom the pandemic may have disrupted their lives in various fields other than health, resulting in a mental health scar that could have an impact afterwards. Thus, SARS-CoV-2 pandemic could be an opportunity to improve mental health services (46–48). Indeed, this translation of immune-inflammatory response into psychiatric illness could improve our knowledge about the pathogenesis mechanisms of these disorders. Altogether, this insight may also help to identify targets for the treatment of inflammation-related neuropsychiatric conditions, and even to develop specific immune-modulator treatments.

## Author Contributions

HB writes the first draft. All authors contributed to and approved the manuscript.

## Conflict of Interest

CL reports personal fees and non-financial support from Janssen-Cilag, Lundbeck, Otsuka Pharmaceutical, and Boehringer Ingelheim in the previous 3 years, outside the submitted work. The remaining authors declare that the research was conducted in the absence of any commercial or financial relationships that could be construed as a potential conflict of interest.

## Publisher's Note

All claims expressed in this article are solely those of the authors and do not necessarily represent those of their affiliated organizations, or those of the publisher, the editors and the reviewers. Any product that may be evaluated in this article, or claim that may be made by its manufacturer, is not guaranteed or endorsed by the publisher.
